# Using a novel smartphone app to track noise and vibration exposure during neonatal ambulance transport

**DOI:** 10.1136/archdischild-2024-327758

**Published:** 2025-01-06

**Authors:** Tom Partridge, Andrew Leslie, Aarti Mistry, Rosalind B Simpson, David E Morris, Donal McNally, John Crowe, Don Sharkey

**Affiliations:** 1 Faculty of Engineering, University of Nottingham, Nottingham, UK; 2 Centre for Perinatal Research, University of Nottingham, School of Medicine, Nottingham, UK; 3 Human Factors Research Group, University of Nottingham Faculty of Engineering, Nottingham, UK

**Keywords:** Neonatology, Technology

## Abstract

**Objective:**

To assess the utility of a bespoke smartphone app to map noise and vibration exposure across neonatal road ambulance journeys.

**Design and setting:**

Prospective observational study of ambulance journeys across a large UK neonatal transport service. Smartphones, with an in-house developed app, were secured to incubator trolleys to collect vibration and noise data for comparison with international standards. A case study exploring alternative routes between hospitals was undertaken.

**Results:**

Over a 12-month period, the app was used to collect data from 1487 interhospital journeys totalling 81 925 km. Noise positively correlated with increasing vehicle speed. Noise exposure never fell below the recommended 45 dB(A) threshold for neonatal patients and exceeded 70 dB(A) for more than 60% of the time. During patient transfers, vibration would be classed as uncomfortable for healthy adults for 68% of journeys. Comparison of 111 journeys on two different routes between the same hospitals demonstrated significantly lower vibration exposure depending on the road type. Safe levels of adult vibration exposure were exceeded on 19% of non-motorway and 3% of motorway journeys between the two hospitals. Vibration and noise levels were significantly higher on concrete compared with asphalt road surface.

**Conclusions:**

It is feasible for neonatal teams to collect detailed route, vibration and noise exposure data using a calibrated smartphone and bespoke app. Collecting large amounts of data and providing live measures to teams could help quantify excessive exposures and guide reduction strategies of these environmental stressors for the benefit of babies, staff and equipment.

**WHAT IS ALREADY KNOWN ON THIS TOPIC**

- Preterm infant transport is associated with increased rates of severe intraventricular haemorrhage. Noise and vibration data collection in the transport environment usually requires additional specialist equipment and personnel.

**WHAT THIS STUDY ADDS**

- A smartphone with a bespoke app allows large amounts of route and exposure data to be collected on the transport environment that could be used to minimise adverse effects of noise and vibration in high-risk infants.

**HOW THIS STUDY MIGHT AFFECT RESEARCH, PRACTICE OR POLICY**

- Novel approaches to collecting large amounts of transport data across different geographical services could be used by transport teams to improve routing decisions to maximise comfort and outcomes for infants.

## Introduction

 Centralised neonatal intensive care has led to the adoption of a policy of postnatal transport for neonatal specialist care.^1 2^ This is widespread across the world and is predicated on transport being acceptably safe for infants.^3^ Multiple studies have found increased rates and severity of intraventricular haemorrhage in transported preterm infants compared with non-transported^4^,^6^ and these are associated with worse neurodevelopmental outcomes.^7 8^ Vibration and noise exposure have been proposed as potential adverse factors contributing to these poorer outcomes,^9^ and a recent Delphi consensus has identified infants’ exposure to these as a research priority.^10^ Small observational studies have measured these in the neonatal transport environment but are restricted by requiring specialist measuring devices, lack of consistent methodology and complex analyses.^11^,^14^

Larger studies across multiple transport services will be needed to understand the association between poor neurological outcomes and transport variables. These will require an easy to implement, consistent and low-cost approach to measuring vibration and noise exposure. This study aimed to investigate whether a standard smartphone with no additional sensors but with a custom-built app could be used by transport team staff to collect data during transport. The secondary aims were to explore the effects of speed, alternative routes and changes in road surfaces on noise and vibration levels and to assess the utility of the data to support approaches to reducing exposure to these.

## Methods

### Setting

Data were collected between 24 October 2018 and 14 October 2019 on ambulance journeys by CenTre Neonatal Transport, which covers an area of 15 811 km^2^ and an estimated population of 4.9 million in the East Midlands region of the UK.^15^ At the time of this study CenTre operated out of bases at Leicester Royal Infirmary and Nottingham City Hospital and used four Fiat Ducato diesel ambulances and four identical neonatal incubator trolleys made from welded tubular steel (ParAid Medical, Solihull, UK) secured longitudinally in vehicles using standard two-part floor locks (Ferno, Cleckheaton, UK).

### Data sources

An app was developed for Android smartphones (Redmi V.5 Plus, Xiaomi, China) that logs sound amplitude, vibration, speed and location data. The smartphones had a built-in accelerometer sampling at up to 200 Hz in front/back(x), side/side(y) and up/down(z) axes. Audio amplitude data were sampled every 20 ms from the smartphone microphone using a built-in method (‘getMaxAmplitude’). Noise data are presented as dB(A). It was not possible to ascertain from the smartphone specification if a dB(A) filter was applied to the noise inputs. However, we have previously reported the smartphone demonstrated a strong linear correlation (r^2^=1.00) with a laboratory microphone recording in dB(A).^16^ No raw audio data were recorded.

Vibration and noise data were mapped to vehicle location and correlated with speed using the global positioning system facility in the smartphone sampled at a rate of 1 Hz. Both the vibration and noise data were calibrated and validated using comparator devices and methods as previously described.^16^

The smartphone was positioned on a horizontal surface on the incubator trolley allowing easy attachment/detachment without impeding clinical activity. Small magnets (N42 Magnets, Magnet Expert, Tuxford, UK) were used to attach the smartphone to the trolley and to secure up to accelerations of approximately 13 g. The smartphone was aligned, so that the measurement axes of its inbuilt accelerometer matched those of the ambulance.

The in-house developed app has a user-friendly interface collecting a small number of data items including details of the trolley and vehicle used, use of emergency driving (blue lights and/or sirens activated) and whether a patient was onboard during the journey. The clinical team placed the activated smartphone on the trolley before departing and were not required to operate it again as data upload to a remote server occurred automatically. Parental consent was not required as no measurements were taken directly from transported infants, care was not altered, and no identifiable patient data were collected.

### Outcomes

There are no evidence or consensus-based guidelines for neonatal transportation vibration and noise against, which data may be benchmarked. Exposure to excess levels of vibration is associated with adverse health effects in healthy adults and in animal studies.^17 18^ We used international standards for vibration exposure in adults, which divide levels of vibration into six bands of perceived comfort level from ‘not uncomfortable’ (<0.32 m/s^2^) to ‘extremely uncomfortable’ (>2 m/s^2^).^19^ The standard recommends that action is taken to reduce exposure to vibration when it exceeds 0.5 m/s^2^ as this is associated with ill health in adults.^19^ For noise exposure, the American Academy of Pediatrics recommends neonatal unit noise levels are kept below 45 dB(A),^20^ as excessive noise can cause physiological instability and impact on growth and development.^21^,^24^

### Variables

**Figure 1:**
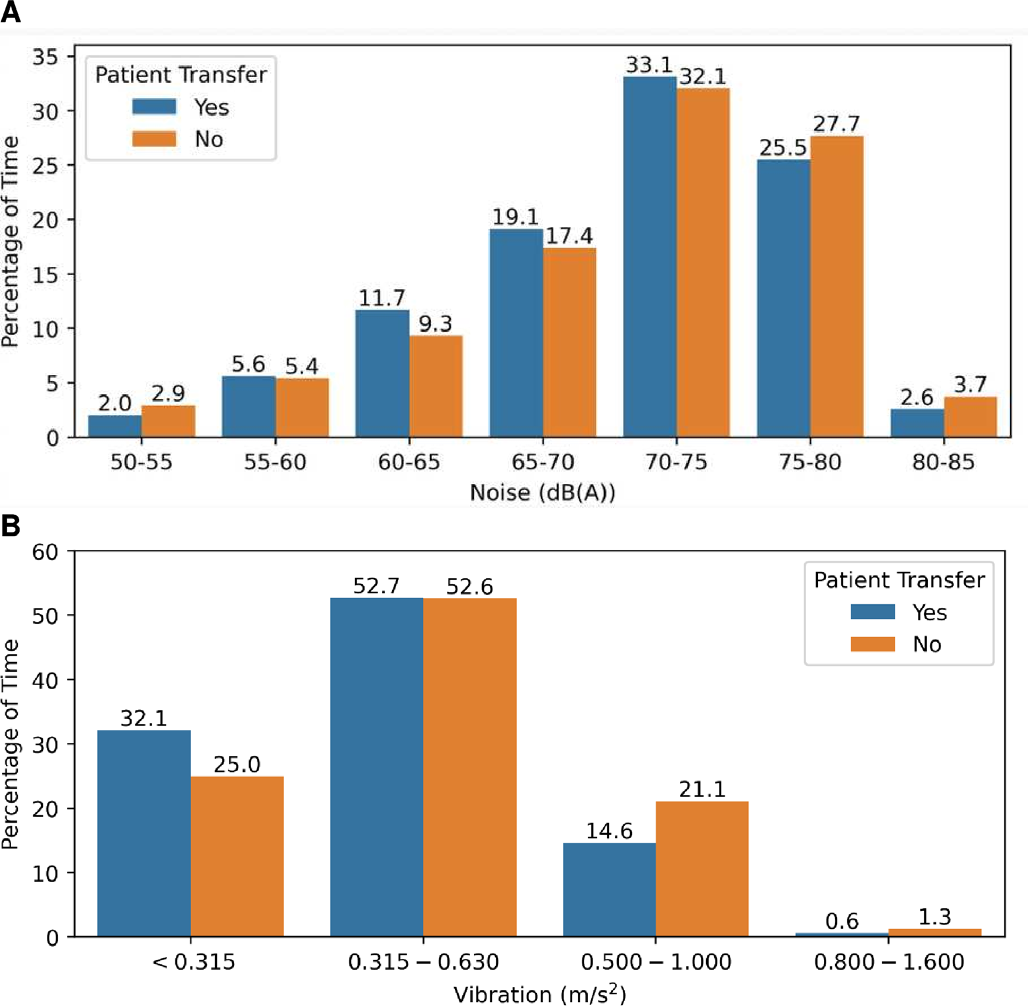
Ambulance journeys when a patient was on-board compared with the non-patient leg of the journey. (**A**) Percentage of journey time on each journey spent at each noise level and (**B**) each ISO-defined vibration comfort level (<0.315 m/s^2^: not uncomfortable; 0.315–0.63 m/s^2^: a little uncomfortable; 0.5–1 m/s^2^: fairly uncomfortable; 0.8–1.6 m/s^2^: uncomfortable; 1.25–2.5 m/s^2^: very uncomfortable; >2 m/s^2^: extremely uncomfortable).^19^

It might be possible to reduce vibration and/or noise exposure by optimising the route taken, and so a proof-of-concept route comparison case study was undertaken. The two most common journeys between two cities were selected as they offered a choice of two main routes: (1) via the motorway to the west or (2) via non-motorway roads to the east (onlinesupplemental figures 1  2). These two routes were compared with understand the impact of routing choices on journey time and patient exposure to noise and vibration.

To understand the impact of road surface on vibration and noise exposure, we identified two distinct sections of an A-road with different surfaces. The main A-road (A46) between the two cities is a dual-carriage way with consistent maximum speed (113 km/hour). It has two types of surface, brushed concrete with expansion gaps spaced at approximately 40 m on the northern section (7.95 km long) and asphalt on the southern section (8.05 km long, online supplemental figure 3). We compared the two types of road surface for noise and vibration.

### Analysis

Average vibration for each journey was obtained by computing the root mean square (RMS) of unweighted vibration at all frequencies in each axis weighted in accordance with ISO 2631-1(19). Average noise was calculated by converting from dB(A) to sound pressure level, computing the RMS, and then converting back to dB(A). Data for the patient and non-patient legs of the journeys were also compared.

Normality of the data was assessed using the Kolmogorov-Smirnov test. Noise and vibration data were normally distributed and two-tailed t-tests were used with a significance level set at p<0.05. Journey time and speed data were not normally distributed, and Mann-Whitney U test was used. Spearman’s rank correlation was used to assess the relationships between increasing vehicle speed and noise/vibration.

## Results

### All journeys

Data were recorded for 1487 journeys totalling 81 925 km travelled over 1331 hours (table 1). The database of all transfers comprised over 946 million data points.

**Table 1:** Summary of all journeys (n=1487)

Journey information	n (%)
Reason for journey (UK-NTG category)	
for uplift of level of care	321 (22%)
repatriation or capacity	333 (22%)
no patient onboard	833 (56%)
Emergency driving used	56 (4%)
Duration of journey (min)*	54 (10–222)
Distance of journey (km)*	55 (5–288)
Average speed of each journey (km/hour)*	15 (3–32)
Average vibration (m/s^2^)*	0.42 (0.26–0.86)
Average noise (dB(A))*	72.4 (62.9–83.5)

Uplift transfers are infants transferred from a neonatal unit that does not offer the level of care required. Repatriation transfers are return of an infant to a neonatal unit closer to home. Capacity transfers are where the infant was born in an appropriate unit but the unit lacks bed space and/or staff.^35^ Emergency driving is use of blue lights and/or sirens and was ascertained in the app by a yes/no question after the transfer was completed.

* Mean (range).

UK-NTG, UK Neonatal Transport Group.

Noise and vibration exposure were compared for journeys with (n=654) and without (n=833) a patient onboard (figure 1). The recommended noise threshold for neonatal patients was exceeded throughout all transfers with no periods under the desired 45 dB(A) limit (figure 1A). For patient transfers, 61.2% of transfer time was spent with noise values ≥70 dB(A).

The percentage of time in each of the vibration level bands demonstrated that vibration levels exceeded the 0.5 m/s^2^ occupational exposure threshold for action 15.2% of the time during patient transfers and 22.4% of the time during non-patient journeys (figure 1B). Overall, 67.9% of patient transfers had average vibration exposure over the comfortable range for healthy adults.

**Figure 2:**
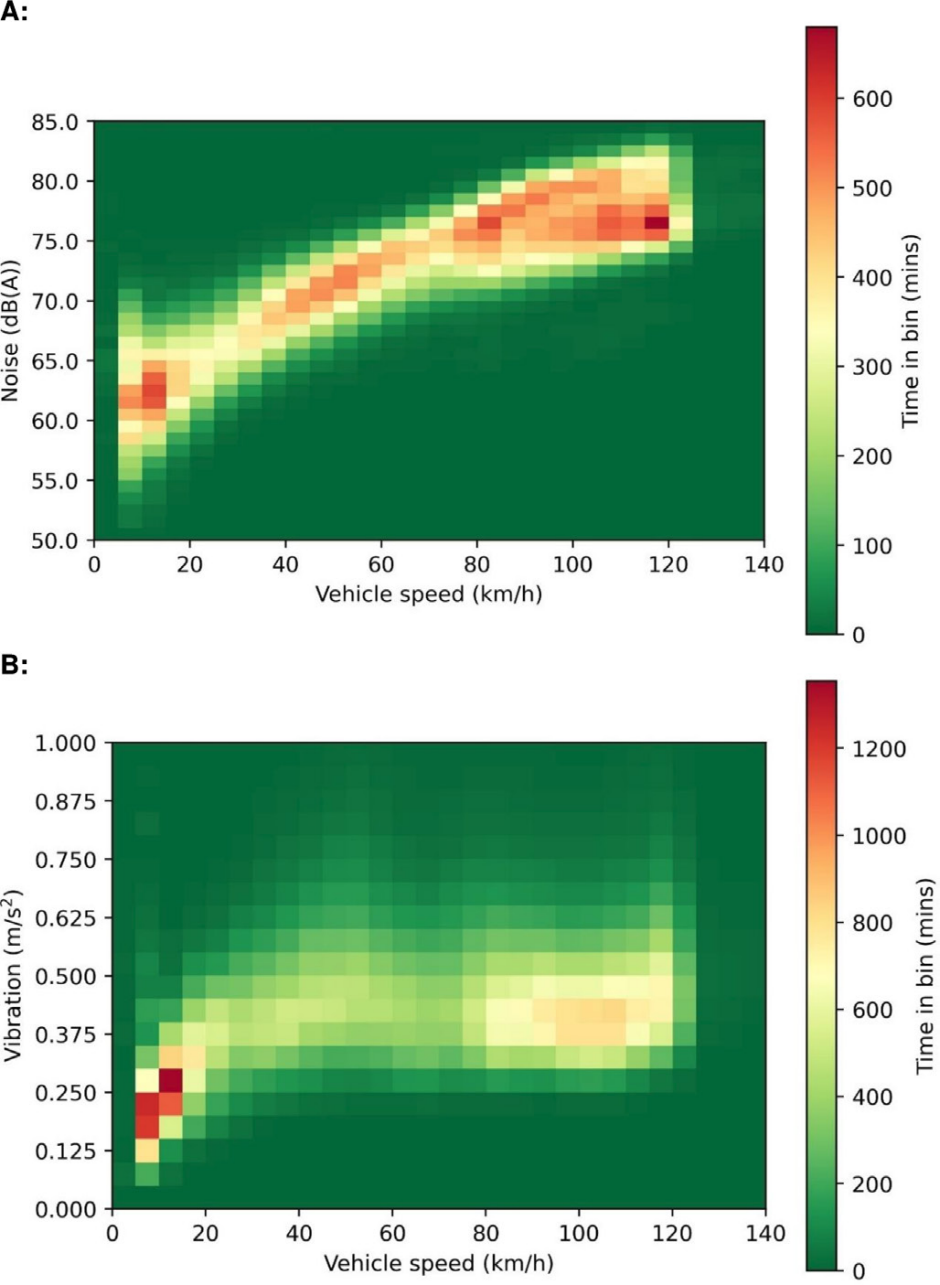
Heat maps for all transfers showing the relationships between vehicle speed and amount of time spent in each speed/noise bin (**A**) and speed/vibration bin (**B**).

Heat maps were generated to identify the relationships between vehicle speed and amount of time spent in each noise or vibration range. As vehicle speed increases, there is an increase in the time spent at higher noise levels (figure 2A) (Spearman’s rank correlation: r_s_=0.39, p<0.001). There was no statistical correlation with vibration and speed (Spearman’s rank correlation: r_s_=−0.02, p=NS). Vibration levels exceed the action threshold for adult workers (0.5 m/s^2^) frequently at all normal driving speeds with vibration levels exceeding the adult exposure limit described as uncomfortable or very uncomfortable (>1.25 m/s^2^) occurring mostly at low speeds (figure 2B).

### Route comparisons

Comparisons were made between journeys on the sections of motorway (n=37) and non-motorway (n=74) for the alternative routes between Nottingham and Leicester. Median journey time was not significantly different between motorway (58 min, IQR 52–61) and non-motorway (54 min, IQR 47–62). Noise levels were also not significantly different between routes. The mean noise level of motorway route journeys was 73.5 dB(A) (95% CI 72.7 to 74.2) and non-motorway-route journeys was 73.5 dB(A) (95% CI 73.0 to 73.9).

**Figure 3:**
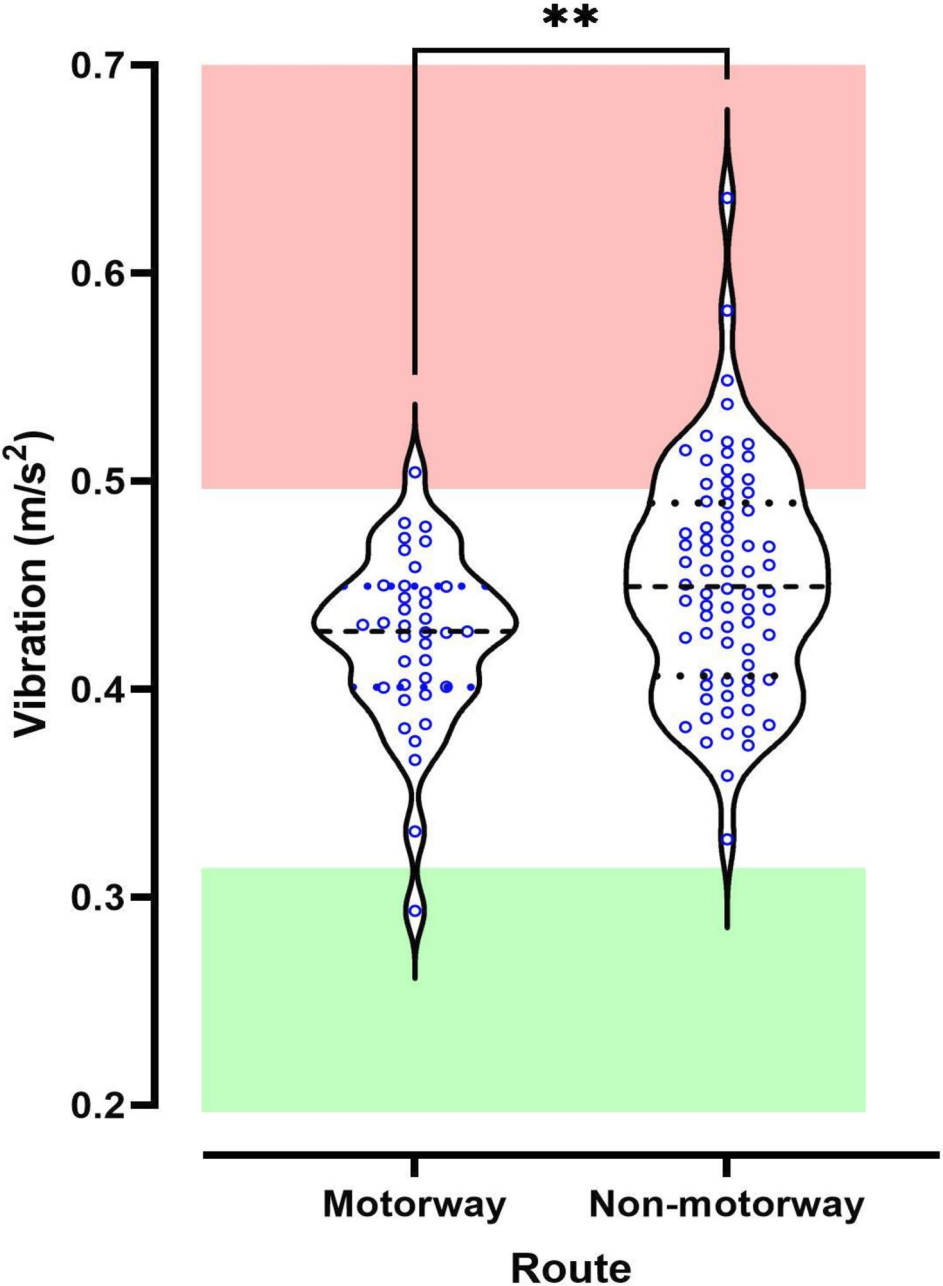
Violin plot of average vibration exposure on two routes, one on the motorway (n=37) and the second on non-motorway roads (n=74), between two neonatal centres in Nottingham and Leicester. Green zone runs up to the upper limit of vibration described as comfortable for adults (0.32m/s^2^), the red zone starts at the lower limit of the legally enforceable vibration exposure allowed for adult workers (0.5m/s^2^).^19^ **p<0.01.

Mean vibration was significantly lower on the motorway route (0.425 m/s^2^, 95% CI 0.412 to 0.439) compared with the non-motorway route (0.442 m/s^2^, 95% CI 0.442 to 0.468) (p<0.01, figure 3). Vibration exceeded adult action threshold (0.5 m/s^2^) for 19% of non-motorway journeys compared with 3% of motorway journeys (p=0.019). Only one journey, on the motorway route, had average vibration below the 0.32 m/s^2^ recommended level.

### Road surface comparison

A total of 71 journeys were made on the same A-road between the two cities. Median speed on the concrete section of road was 99 km/hour (IQR 97–101) and on the asphalt section was 103 km/hour (IQR 101–105) (p=0.04). Mean noise levels were significantly higher for the concrete section (77.8 dB(A), 95% CI 77.76 to 77.83) compared with the asphalt section (76.1 dBA, 95% CI 76.05 to 76.14) (p<0.001). Vibration was also significantly higher on the concrete section (0.452 m/s^2^, (95% CI 0.443 to 0.461) compared with the asphalt section (0.398 m/s^2^, (95% CI 0.392 to 0.404) (p<0.001).

## Discussion

We have demonstrated the feasibility of using inexpensive, off-the-shelf smartphones to collect large amounts of noise and vibration data during ambulance transfer. This study demonstrates that high levels of noise and vibration are experienced routinely during ambulance transport and that crucially, these are modifiable with alterations to speed and route. It is unclear if the adverse outcomes associated with neonatal transfer are caused in part by exposure to high levels of noise and vibration due to a lack of large population-based studies linked with these measures. Our approach demonstrates the ability to collect these measures at scale, which could link with patient outcomes.

### Noise exposure

In keeping with other studies over the last 30 years, we have shown that noise exposure during neonatal transport remains high.^911 14 25^,^28^ Average levels over 70 dB(A) are present for over 60% of journeys, exceeding reported noise levels in neonatal units.^22^,^24^ Noise of this intensity has been shown to have adverse effects on infants including transient cardiorespiratory changes and pain behaviours.^21^ Up to 10% of preterm infants have hearing impairment, far higher than the estimated 0.1% of the general population.^29^ Neonatal hearing loss is multifactorial and it is unclear what is the role of excessive noise over prolonged periods as observed in this study. In our case study of road surfaces, we found a reduction in sound levels on asphalt compared with concrete, supporting the hypothesis that route guidance could help reduce the infant’s exposure to excessive sound. A recent mannequin study has suggested potential benefit from active noise cancellation.^27^

### Vibration exposure

Vibration levels exceeding occupational standards for adults are common at all speeds during neonatal ambulance journeys.^12 13 26 30^ In this study, the measured vibration levels at speeds >20 km/hour frequently exceeded the 0.32 m/s^2^ maximum occupational exposure level.^19^

The comparison of patient/non-patient journeys shows that driving behaviour is beneficially modified when a patient is present, suggesting that driver behaviour is modifiable. Provision of live in-transit data on ride quality may help optimise comfort for all occupants.^31^ Reducing vibration of equipment and the ambulance could extend their lifespan or reduce breakdowns.

### Optimal routing

We have shown that it is possible to add information on impact of route choices on the environment of the patient and this approach is both inexpensive and scalable, allowing it to be adopted by other transport teams. In the example given, one route was superior for vibration and noise at the cost of a 4 min prolongation of the journey, a time addition unlikely to be of clinical significance. Data could inform route choice and allow driving adaptations to improve comfort. Minimising vibration could also help prevent adverse events to the patient,^32 33^ for example, in extremely preterm ventilated infants, the movement of their endotracheal tube by a few millimetres could result in suboptimal positioning or dislodgement and so become a medical emergency.

The app produces a postjourney summary that can be shared with the team who undertook the transfer, summarising vibration and noise data overlaid on the route map (online supplemental figure 4). It is possible that provision of automated rapid feedback after every journey will promote positive route choices or driving behaviours.

The absolute reductions seen in vibration and noise with route optimisation are relatively small but may be cumulatively important in improving patient comfort and reducing the risks of transport. This is the first study to offer patient-focused analysis of route choices and suggests possibilities for further investigation in this area.

### Limitations

The main limitation of this study is that the smartphone is mounted on the trolley, so will not completely reflect the infant’s environment. There are data that suggest that vibration is 2–4 times higher at the infant head than at the trolley.^12^ Noise experienced by the infant may also be different from that measured at the trolley. Equipment inside the incubator, such as gas flow through breathing circuits, may add noise and external noise may be modified by the incubator enclosure. We did not account for noise attenuation approaches such as ear defenders, which may help reduce levels. The smartphone noise measurement capability is limited at levels >90 dB(A), so peaks may be underestimated. Only one model of transport incubator trolley and road ambulance were studied and there may be differences with other equipment configurations and vehicles.

Any vibration or noise differences due to emergency driving could not be elicited from the data. Lights and sirens may be used briefly or intermittently during a journey and the app did not allow these episodes to be isolated.

Only one model of smartphone was used in this study and the data obtained were validated in laboratory conditions.^16^ It cannot be assumed that other models of smartphone are equally accurate or sample at the same rates.

The majority of UK neonatal transports are by road ambulance with very few by air.^34^ Journeys by air will have different vibration and noise characteristics from ground journeys.^11 26^

The absence of evidence-based standards for neonatal noise and vibration exposure imposes limitations on the interpretation of the results. It remains unclear whether the associated adverse outcomes for transported infants are caused, at least in part, by noise and vibration exposure during transport. Further work is needed to provide a detailed understanding of pre and post-transport condition of infants and to match this with reliable detailed data about the transport environment.

This study has not considered the effects of vibration exposure on the staff of transport services. Many UK transport services have staff who work wholly for transport and who have as yet unmeasured occupational exposure risks.

## Conclusion

We have shown that a smartphone with a bespoke app was easily adopted by clinical teams and may be used to collect reliable and detailed noise and vibration data on neonatal interhospital journeys. The data have been used to show sample comparisons between routes, road surface type and driving styles, all of which may be factors that could be further investigated for their potential to improve safety and comfort for transported infants and the teams caring for them. This novel approach is inexpensive, scalable and offers an opportunity to expand to include other transport services, transport modes and ultimately develop evidence-based standards and outcome measures aimed at improving transport safety.

## Supplementary material

10.1136/archdischild-2024-327758 online supplemental file 1

10.1136/archdischild-2024-327758 online supplemental file 2

10.1136/archdischild-2024-327758 online supplemental file 3

10.1136/archdischild-2024-327758 online supplemental file 4

## Data Availability

Data are available upon reasonable request.
