# Supplementary material for: Using a novel smartphone app to track noise and vibration exposure during neonatal ambulance transport
Source: Arch Dis Child Fetal Neonatal Ed. 2025 Jan 6;110(4):e327758. doi: 10.1136/archdischild-2024-327758 (PMC12229049; doi:10.1136/archdischild-2024-327758)

Supplemental Figure 2 – Route choices between Nottingham and Leicester – via the motorway to the west or the A-Road to the east.

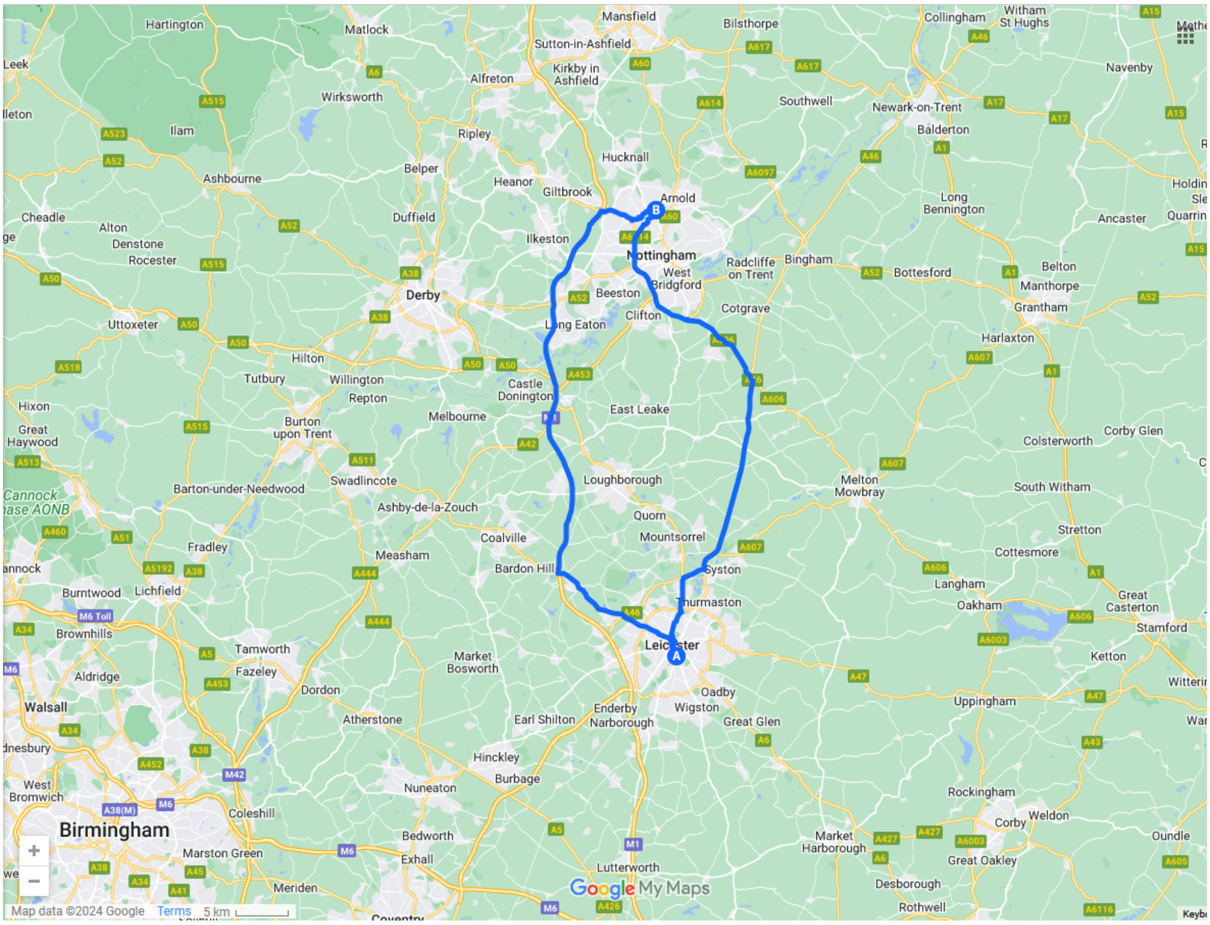

Supplement: online supplemental file 2 [file fetalneonatal-110-4-s002.pdf]
