# Supplementary material for: Using a novel smartphone app to track noise and vibration exposure during neonatal ambulance transport
Source: Arch Dis Child Fetal Neonatal Ed. 2025 Jan 6;110(4):e327758. doi: 10.1136/archdischild-2024-327758 (PMC12229049; doi:10.1136/archdischild-2024-327758)
Supplement: online supplemental file 3 [file fetalneonatal-110-4-s003.pdf]

Supplemental Figure 3 – Transition from concrete road surface to asphalt on A-Road.

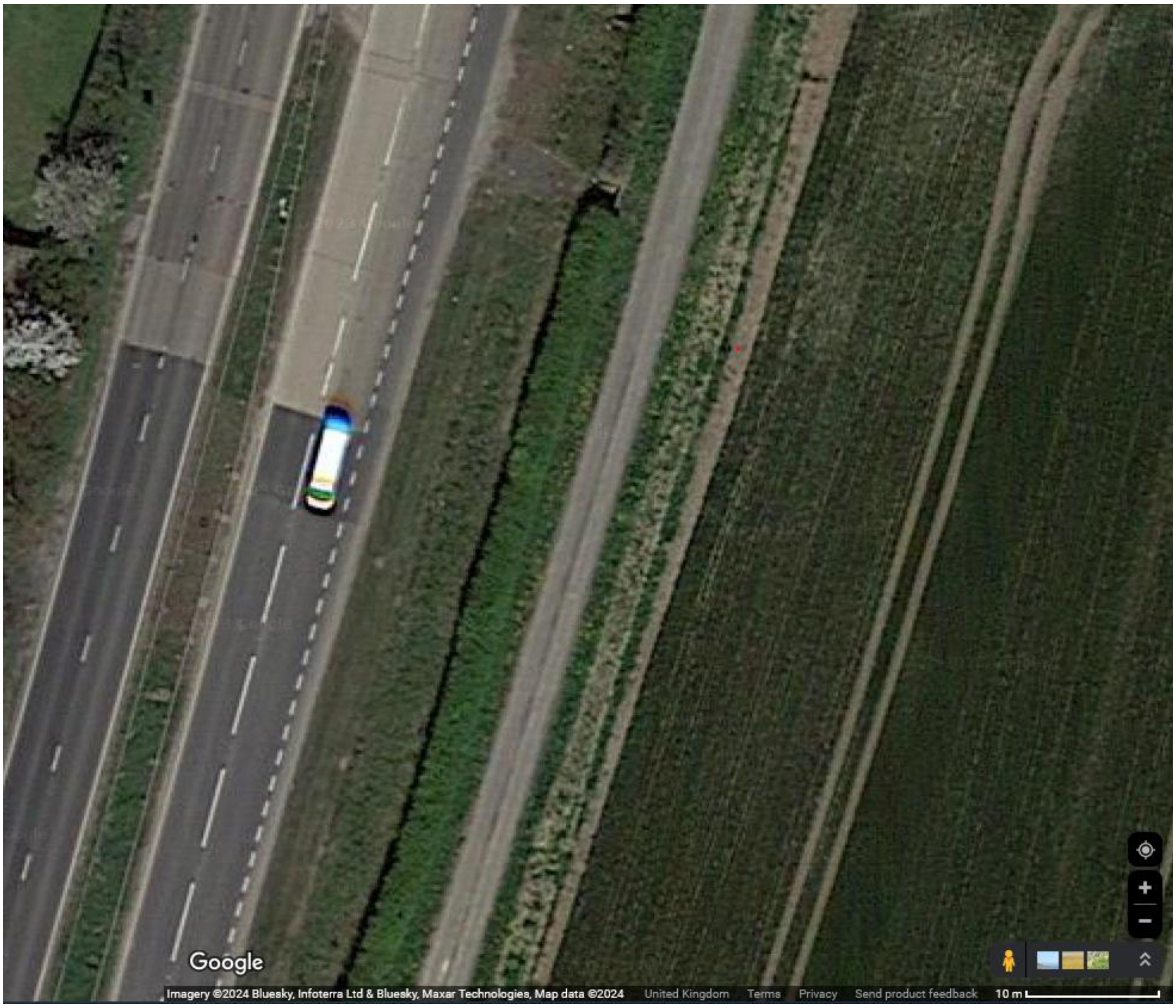

Location points:

A46 start concrete 52.849362, -1.032789, end 52.779006, -1.047635

A46 start asphalt 52.779006, -1.047635, end 52.699575, -1.096045
