# Supplementary material for: Using a novel smartphone app to track noise and vibration exposure during neonatal ambulance transport
Source: Arch Dis Child Fetal Neonatal Ed. 2025 Jan 6;110(4):e327758. doi: 10.1136/archdischild-2024-327758 (PMC12229049; doi:10.1136/archdischild-2024-327758)
Supplement: online supplemental file 4 [file fetalneonatal-110-4-s004.pdf]

Recording Date: 24-10-2018 08:53

Journey Start	Journey End	Patient transported
09:02	09:59	No
Nottingham City	Leicester Royal	

Duration	Distance	Median Speed	Av. WBV	Av. Noise
57.5 minutes	50.9 km	14.5 km/h	0.5 m·s^{-2}	74.4 dB(A)

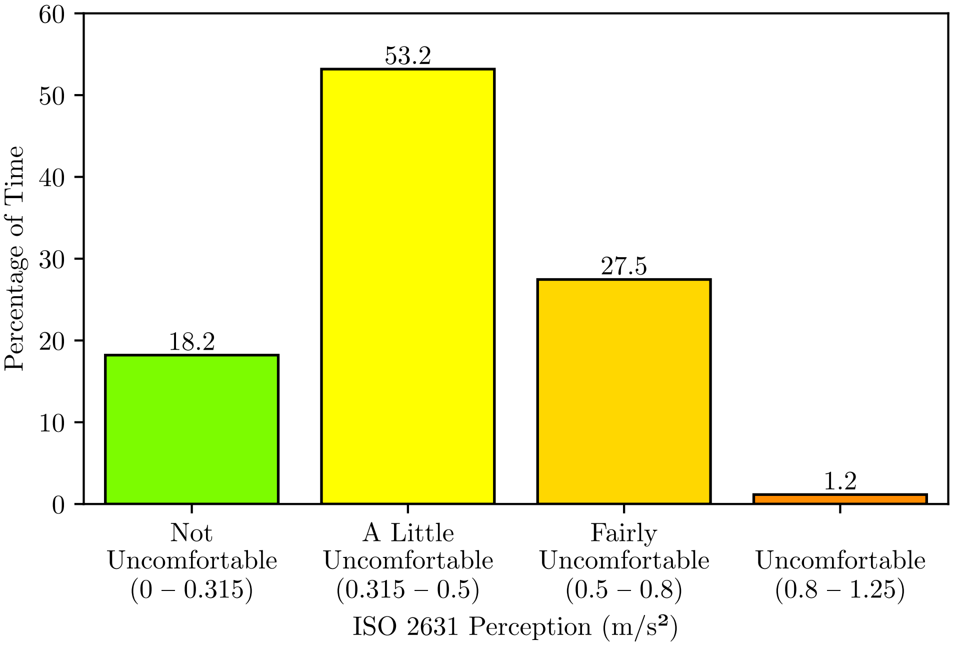

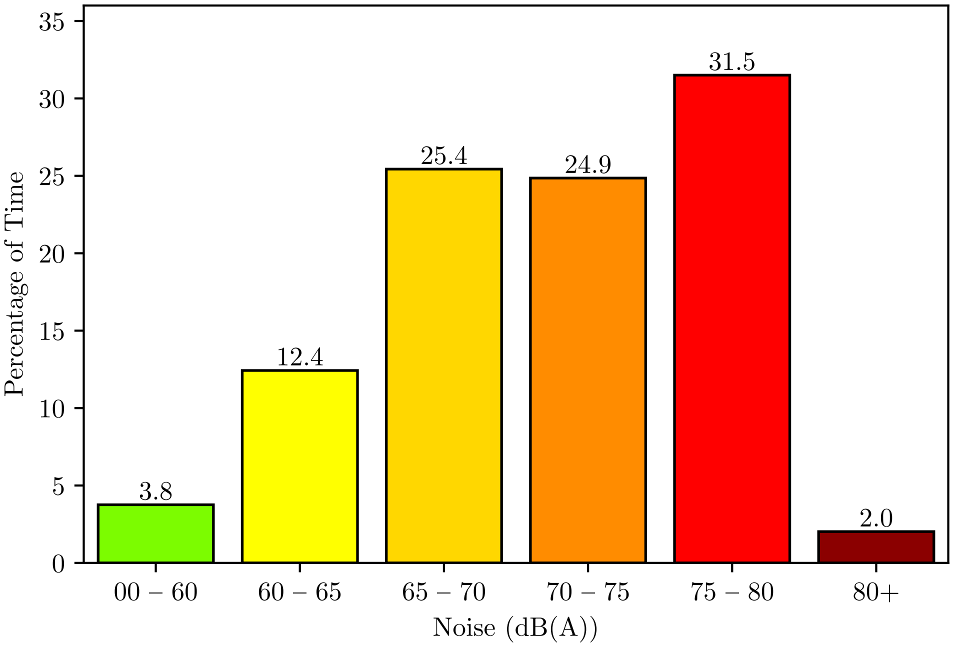

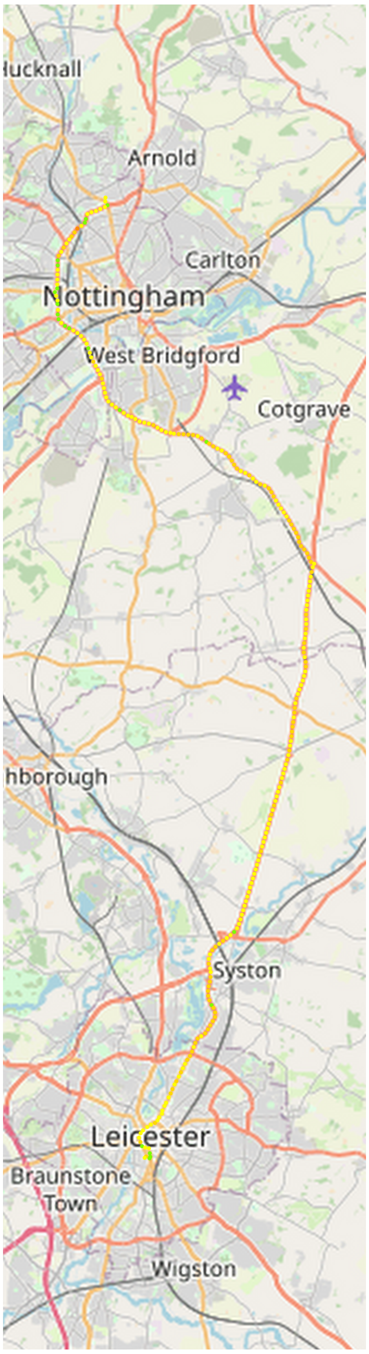

Average WBV along route.

WBV Perception
- Extremely Uncomfortable
- Very Uncomfortable
- Uncomfortable
- Fairly Uncomfortable
- A Little Uncomfortable
- Not Uncomfortable

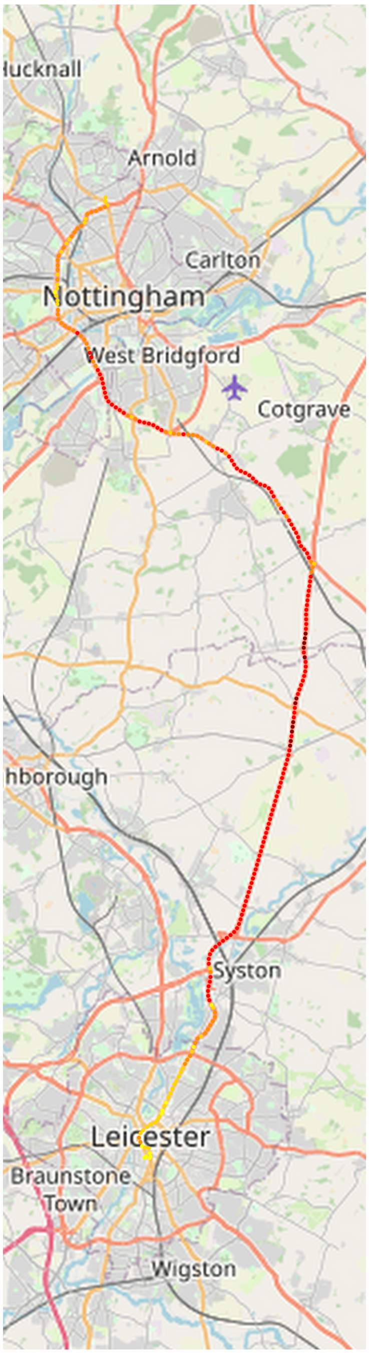

Average Noise along route.

A-weighted Noise Level
- 80+
- 75 – 80
- 70 – 75
- 65 – 70
- 60 – 65
- 00 – 60
